# A high serum level of eotaxin (CCL 11) is associated with less radiographic progression in early rheumatoid arthritis patients

**DOI:** 10.1186/ar2381

**Published:** 2008-03-02

**Authors:** Silje W Syversen, Guro L Goll, Espen A Haavardsholm, Pernille Bøyesen, Tor Lea, Tore K Kvien

**Affiliations:** 1 Department of Rheumatology, Diakonhjemmet Hospital, Oslo, Norway; 2 Faculty of Medicine, University of Oslo, Oslo, Norway; 3 Institute of Chemistry, Biotechnology and Food Science, Norwegian University of Life Sciences, Ås, Norway

## Abstract

**Introduction:**

Prognosis in rheumatoid arthritis (RA) is difficult to assess. The aim of this study was to examine whether serum levels of a spectrum of cytokines were predictive of radiographic progression in early RA patients.

**Methods:**

A total of 82 early RA patients (disease duration < 1 year) were followed for 12 months. Clinical assessments, X-rays of hands and magnetic resonance imaging (MRI) of the dominant wrist were assessed at baseline and after 3, 6 and 12 months. The X-rays were scored according to the van der Heijde modified Sharp score (vdHSS). Cytokine analyses were performed with multiplex technology. Associations between cytokines and radiographic progression were examined by logistic regression.

**Results:**

In all, 49% of the patients developed radiographic progression. The median (interquartile range (IQR)) baseline eotaxin level (pg/ml) was significantly lower in patients with (193 (119 to 247)) than without progression (265 (166 to 360)). In the univariate logistic regression analyses, eotaxin was negatively associated to radiographic progression, and this association was maintained in the multivariate model with an odds ratio (OR) (95% confidence interval (CI)) for progression of 0.58 (0.41 to 0.82) per 50 pg/ml increase in eotaxin level. None of the other measured cytokines showed any association to radiographic progression.

**Conclusion:**

This study raises the hypothesis that high serum levels of eotaxin predict less radiographic progression in early RA patients.

## Introduction

Rheumatoid arthritis (RA) is a chronic inflammatory disease characterized by synovitis, progressive erosions and cartilage destruction. Early and aggressive treatment is now widely recommended to suppress inflammation and limit cartilage and bone loss. The disease course, however, is often unpredictable, with pronounced intra-individual variations.

Markers that directly reflect the inflammatory activity in the joint would be useful clinical tools. Cytokines are local protein mediators known to be involved in almost all important biological processes. The RA synovium represents one of the best studied cytokine networks of human autoimmune diseases [1,2]. Recognition of cytokines as mediators of inflammation and cartilage and bone destruction has greatly improved the treatment of RA patients.

New technology permits simultaneous analysis of a panel of cytokines in a low volume biological sample [3]. Whether serum measurements of cytokine activity can contribute in assessing RA prognosis is currently unclear, and can only be tested in well characterized longitudinal cohorts. The aim of this study was to explore the associations between the serum levels of a broad spectrum of T- and B-cell derived cytokines, growth factors and chemokines and 1-year radiographic progression in early RA patients.

## Materials and methods

### Patients

A total of 84 consecutively enrolled RA patients with disease duration of less than 1 year were followed for 12 months [4]. Clinical examination, X-rays of hands, magenetic resonance imaging (MRI) of the dominant wrist and laboratory analyses were assessed at baseline and after 3, 6 and 12 months. The patients received treatment according to clinical practice. Disease-modifying antirheumatic drugs (DMARDs) were used by 77.4% of the included patients at baseline. During the study, DMARDs were used by 91.8% of the patients and anti-tumor necrosis factor (TNF)α drugs by two patients (2.6%). The regional ethics committee evaluated the study, and all included patients gave informed consent.

### Laboratory analyses

Serum samples were stored at -70°C. Erythrocyte sedimentation rate (ESR) was measured by an in-house Westergren method, antibodies to cyclic citrullinated peptide (anti-CCP) were analyzed by a second generation ELISA kit (INOVA Diagnostics Inc, San Diego, USA), IgM rheumatoid factors by an in-house ELISA and high sensitivity C-reactive protein (hsCRP) was measured by nephelometry (Dade Behring, USA). Cytokine analyses were performed with multiplex fluoroimmunoassay technology (Biosource Cytokine 25-plex from Luminex Corporation, Austin, USA). The multiplex assay system has been validated against the traditional ELISA sandwich assay for cytokines and found to perform very reliably [5]. Measured cytokines were: eotaxin, TNFα, interferon (IFN)γ, IFNα, macrophage inflammatory protein (MIP)1-α, IP10 Human interferon-gamma (IFN-gamma)-inducible protein 10, monokine induced by gamma interferon (MIG), monocyte chemotactic protein (MCP)1, granulocyte-macrophage colony-stimulating factor (GM-CSF), MIP1β, IL1β, IL1-ra, IL2, IL2R, IL4, IL5, IL6, IL7, IL8, IL10, IL12, IL13, IL15, IL17 and RANTES (regulated on activation normal T-cell expressed and secreted).

### Acquisition and assessment of MRIs and conventional radiographs

X-rays of the hands were scored according to the van der Heijde modified Sharp score (vdHSS) by a trained observer (PB). In each hand, 16 joint areas were scored for erosions (score range for each area 0 to 5) and 15 areas were scored for joint space narrowing (range of score from 0 to 4 for each joint area), giving a possible maximum score of 280 units. MRI of the dominant wrist was performed using a Sigma 1.5 Tesla MRI scanner (General Electric, Milwaukee, WI, USA). The MRI sequences and scoring in this study have previously been described in detail [6]. Images were scored according to the semi-quantitative RA magnetic resonance imaging score (RAMRIS) [7] by a trained observer (EAH).

### Statistical methods

Statistical analyses were performed using SPSS 14 (SPSS Inc, Chicago, IL, USA). Data were presented by median (interquartile range (IQR)) because of skewed data. Correlations were assessed by Spearman's test of rank correlation. Comparisons between groups were examined by the Mann-Whitney U test. Uni- and multivariate logistic and linear regression analyses were performed to examine the predictive potential of the cytokines. Clinically important covariates were included in the multivariate analyses if univariate analyses revealed a p value < 0.25. All tests were two-sided and p values below 0.05 were considered to be statistically significant. Correction for multiple testing was not performed.

## Results

The baseline characteristics of the cohort with averages given as median (IQR) were as follows: age 58 (47 to 67) years, disease duration 107 (77 to 188) days and 28-joint Disease Activity Score (DAS28) 4.2 (3.0 to 5.1). A total of 77.4% of patients were female, 44% were IgM rheumatoid factor (RF) positive and 55% anti-CCP positive. Associations between baseline serum level and radiographic progression were examined for all the 25 cytokines. We will only focus on the eotaxin results in this paper as none of the other cytokines showed any association to radiographic progression.

Median (IQR) baseline eotaxin level (pg/ml) was 214.0 (137.2 to 323.3), mean (SD) level 242 (163) (pg/ml). Suggested normal values from the manufacturer were 72 to 248 pg/ml. There was no correlation between baseline eotaxin level and measures of disease activity measured by DAS28 and acute phase reactants. A correlation coefficient of 0.27 (p 0.02) was found between eotaxin and anti-CCP.

In all, 49% of the patients developed radiographic progression (increase in total vdHSS score ≥ 1 unit) during follow-up. Median (IQR) baseline eotaxin level (pg/ml) was significantly (p = 0.007) lower in patients with (193 (119 to 247)) than without radiographic progression (265 (166 to 360)). The baseline eotaxin level was moderately and significantly negative correlated (r = -0.35) to a change in vdHSS during follow-up. In the univariate logistic regression analyses eotaxin was significantly (p < 0.05) associated to radiographic non-progression, with an odds ratio for progression of 0.70 per 50 pg/ml increase in eotaxin. We controlled for other known predictors of radiographic progression (ESR, CRP, baseline vdHSS, anti-CCP, IgM RF) and age and gender in a multivariate logistic regression model. We also included RAMRIS bone marrow edema score > 2 in the model based on previous findings in the same cohort of patients [4]. In the final logistic regression model, the adjusted odds ratio (OR) (95% confidence interval (CI)) for progression was 0.58 (0.41 to 0.82) per 50 pg/ml increase in eotaxin level (Table 1). Hence, in this study, an increase in the baseline eotaxin level of 50 pg/ml corresponded to a 42% decrease in the odds of radiographic progression. The range of eotaxin is 50 to 500 pg/ml. Bone marrow edema on MRI, male gender and disease activity at baseline also independently predicted radiographic progression. The accuracy of the model to predict radiographic progression was 71%. In the regression analyses, eotaxin showed linearity without any obvious cut-off value for discrimination between progression and non-progression. In a receiver operating characteristic (ROC) analysis the value of 214 (corresponding to the median value) gave the highest value for the combination of sensitivity (0.6) and specificity (0.6). The area under the curve was 0.69. When eotaxin was entered into the logistic regression as a dichotomized variable according to the ROC analysis, the OR for progression with a low eotaxine was 3.6 (CI 1.1 to 11.9; Table 1). The accuracy of this model is 64%.

**Table 1:** Predictors of 12 months radiographic progression

	Eotaxine as a continous variable		Eotaxine as a dichotomized variable	
	OR (95% CI)	p Value	OR (95% CI)	p Value

Baseline variable:				
Eotaxin (per 50 pg/ml)	0.6 (0.4–0.8)	< 0.01		
Eotaxine (low < 214 vs high > 214)			3.6 (1.1–11.9)	0.04
DAS28	1.6 (1.0–2.4)	0.05		
RAMRIS bone marrow edema score > 2	3.4 (1.0–11.2)	0.05	3.7 (1.2–11.7)	0.03
Male	6.9 (1.6–30.5)	0.01	3.5 (0.9–13.6)	0.06

DAS28, 28-joint Disease Activity Score; OR, odds ratio; RAMRIS, rheumatoid arthritis magnetic resonance imaging score.

To gain statistical power, a multivariate linear regression model was also applied and gave similar results (data not shown).

## Discussion

RA patients express increased serum levels of several cytokines, but the evidence for a clinical relevance of such measurements in the assessment of prognosis is limited. In this 1-year prospective study of 84 early RA patients, we found that a high serum level of eotaxin in the early disease course was associated with less subsequent radiographic progression. The association between eotaxin level at baseline and radiographic progression remained strong when adjusting for other known predictors of joint destruction. Serum levels of the other tested cytokines in this study were not associated with radiographic progression.

Chemokines are suggested to be important in RA pathology and elevated levels have been measured in the synovium and serum of RA patients [8-10]. Hueber *et al*. found elevated serum levels of chemokines to be the most evident difference between early RA patients and controls [10]. Eotaxin (chemokine C-C motif ligand 11 (CCL11)), a member of the CC chemokine family, recruits eosinophils to sites of inflammation, particularly in allergic diseases and asthma [11]. It is produced by lymphocytes, eosinophils and monocytes/macrophages, and interacts with C-C chemokine receptor 3 (CCR3) [11]. This receptor is expressed by T-lymphocytes, eosinophils, basophils, dendritic cells, and a recent study has also suggested expression of CCR3 on osteoclasts [11-13].

A Korean study has suggested a link between eotaxin gene polymorphisms and RA [14]. Two recent studies have shown increased eotaxin in the plasma of juvenile idiopathic arthritis (JIA) and serum of RA patients compared to normal controls [10,15], and it has been suggested that eotaxin may be one of several cytokines denoting low activity in JIA joint disease [15]. The possible expression of the CCR3 receptor on osteoclasts and the suggested involvement in osteoporosis might indicate a role in bone resorption [12,13].

The role of eotaxin in RA patients and thus the pathophysiological impact of the negative association to radiographic progression, however, are currently unclear. One may speculate whether high eotaxin levels denote a more T-helper (Th) 2-type response in individual RA patients, possibly protecting against joint damage.

Our study has some limitations. Patients were treated according to clinical judgment before inclusion and during the study. The 1-year follow-up could be insufficient to reveal an association between progression and serum levels of any of the other tested biomarkers. We cannot exclude that the association between eotaxin levels and radiographic progression is an arbitrary finding due to multiple testing or an unknown confounder. The p values, however, were far below 0.01, despite the relatively small number of patients included.

One can question whether measurements of cytokines in serum reflect the cytokine levels in the joint; however serum measurements of cytokines might regardless be clinically useful if associated to important patient outcome. To our knowledge this is the first prospective study to explore the associations between radiographic progression and a broad spectrum of cytokines.

## Conclusion

This study raises the hypothesis that low serum levels of eotaxin predict radiographic progression in early RA patients. It is, however, critical that the relationship is confirmed in an independent cohort of patients, preferably with longer follow-up time.

## Authors' contributions

SWS participated in study design and coordination, analyzed and interpreted the data and drafted the manuscript, GLG participated in study design and coordination, assisted in drafting the manuscript, and EAH and PB participated in study design and coordination, read and interpreted the MRIs and X-rays. TL analyzed the cytokines, TKK designed the study, assisted in interpretation of statistical analyses and drafting of the manuscript. All authors read and approved the final manuscript.

## Abbreviations

CCL = chemokine C-C motif ligand; CCP = cyclic citrullinated peptide; CCR = C-C chemokine receptor; CI = confidence interval; CRP = C-reactive protein; DAS28 = 28-joint Disease Activity Score; DMARD = disease-modifying antirheumatic drug; ELISA = enzyme-linked immunosorbent assay; ESR = erythrocyte sedimentation rate; GM-CSF = granulocyte-macrophage colony-stimulating factor; hsCRP = high sensitivity CRP; IFN = interferon; IL = interleukin; IQR = interquartile range; JIA = juvenile idiopathic arthritis; MIG = monokine induced by gamma interferon; MIP = macrophage inflammatory protein; MRI = magnetic resonance imaging; OR = odds ratio; RA = rheumatoid arthritis; RAMRIS = RA magnetic resonance imaging score; RANTES = regulated on activation normal T-cell expressed and secreted; RF = rheumatoid factor; ROC = receiver operating characteristic; TNFα = tumor necrosis factor α; vdHSS = van der Heijde modified Sharp score.

## Competing interests

The authors declare that they have no competing interests.
